# Alcohol use disorder among prisoners in Debre Berhan prison, Ethiopia: a cross-sectional study

**DOI:** 10.1186/s13011-020-00270-w

**Published:** 2020-04-03

**Authors:** Yohannes Gebreegziabhere Haile, Kaleab Berhanu Kebede, Asnake Limenhe, Kassahun Habatmu, Atalay Alem

**Affiliations:** 1 Department of Nursing, Debre Berhan University, Debre Berhan, Ethiopia; 2 Department of Psychiatry, College of Health Sciences, Addis Ababa University, Addis Ababa, Ethiopia; 3 School of Psychology, College of Education and Behavioral Studies, Addis Ababa University, Addis Ababa, Ethiopia

**Keywords:** Substance use, Alcohol use disorder, Prevalence, Associated factors, Prison, Ethiopia

## Abstract

**Background:**

Several studies reported that history of alcohol use among prisoners is higher than the prevalence in the general population. Criminality is found to be associated with alcohol use disorder (AUD) in previous studies. In Ethiopia, there is limited information on the prevalence and associated factors of AUD among prisoners. Therefore, this study aimed to assess the prevalence and associated factors of AUD among prisoners of Debre Berhan Prison.

**Methods:**

A cross-sectional survey was conducted to assess history of AUD among prisoners at Debre Berhan Prison, before imprisonment. We selected 347 prisoners with a systematic sampling technique and interviewed using Alcohol Use Disorder Identification Test (AUDIT) to screen for AUD in May 2017. Data entry was done using Epi-Data version 3.1 software, and bivariate and multivariate analyses were done using Stata version 13 software. Crude and adjusted odds ratios, with 95% confidence intervals and *p*-values are reported.

**Results:**

About six out of ten prisoners (59.1%) had AUD before imprisonment. Factors associated with increased odds of AUD were perception that the current offence is related to using substances (AOR = 4.2; 95% CI = 2.3, 7.8), and family history of substance use (AOR = 8.7; 95% CI = 1.7, 44.9). Being married had lower odds of AUD compared to the unmarried (AOR = 0.5; 95% CI = 0.2, 0.9).

**Conclusion:**

We found that the prevalence of AUD 1 year before imprisonment in this population is high. AUD is found to be associated with a family history of substance use and perception that the current offence is related to using a substance. We recommend community-based study with different kind of study designs to see the relationship between AUD and crime for planning interventions.

## Background

The burden of mental and substance use disorders is high globally, and it is particularly worse in alcohol-related problems, according to the World Health Organization (WHO), worldwide three million deaths (5.3% of all deaths) every year resulted from harmful use of alcohol [1]. In support of this, the WHO global burden of diseases 2016 report showed that alcohol was the seventh leading risk factor for both deaths and disability-adjusted life years (DALYs) [2]. In terms of year’s lost due to disability (YLDs), in 2010 mental and substance use disorders were the leading (22.9% of the world YLDs or 175.3 million YLDs) [3]. In the same document, it was reported that about 184 million DALYs worldwide were due to mental and substance use disorders (7.4% of all the DALYs) and ranked 5th among the top 10 causes of DALYs. The WHO global burden of disease 2010 report also showed that 9.6% of DALYs were due to mental and substance use disorders secondary to alcohol use.

Every year 6.2 l of pure alcohol is consumed worldwide per person of age greater than 15 years [4]. In Ethiopia, the consumption of alcohol was reported to be 4.2 l per person of age older than 15 years per year, in 2010 [5]. WHO sets a target of reducing the harmful use of alcohol by 10.0% by the year 2025 [4]; we believe that this may not be achieved without including the prison population as part of a targeted population group for intervention.

According to the international center for prison studies report in 2013, over the last 15 years before 2013, the world population had increased by over 20%, but the world prison population during the same period increased by 25–30% [6]. The same report disclosed that as of the beginning of October 2013, more than 11 million people in the world were either in prison, in pre-trial, or in administrative detention; which translates to 155 people per 100, 000 population. The same report showed that in 2013 there were about 112,361 prisoners in Ethiopia (this is equivalent to 136 prisoners per 100,000 people) [6].

Several studies have reported that crime and substance use have direct relationship [7–9]. In addition, alcohol use has been reported to be associated with intimate partner violence [10]. One of the reasons why people use substance is to increase their confidence to commit crime [11]. In support of this, one study found that 10% of homicide offenders claimed that the offense took place while they were under the influence of a substance [12].

Alcohol is among the top five list of drugs abused among prisoners, either in prison or before imprisonment [13–19]. Compared to the general population alcohol use was reported to be twice as common in prisoners [20]. The prevalence of any substance use among prison populations ranged from as low as 20.1% to as high as 95.5% [18, 21–24]. Prevalence ranging from 10.7 to 70.0% was reported for alcohol use disorder (AUD) [13, 17–19, 21–28]. A systematic review of studies from the United Kingdom reported that 13 to 86% of prisoners at a different stage of imprisonment (remanded and sentenced) had alcohol dependence or AUD [29]. A 20-year study to see the trended of AUD, from 1985 to 2006, showed an increase in the prevalence of AUD in male prisoners from 41% in 1985 to 52% in 2006 [30]. This indicates that the trend of AUD while in prison increases with time.

On the other hand, the prevalence of substance use disorder (SUD) among prisoners before imprisonment was higher than both from use in prison and use in the general population, which may indicate association between crime and substance use. Studies in prisoners have shown that prevalence ranged from 50 to 88% for general substance use and 13 to 86% for AUD before imprisonment [11, 12, 16, 31–45]. A recent systematic review of about 24 studies from 10 different countries reported prevalence of AUD ranging from 16 to 51% among newly incarcerated male prisoners, with a pooled prevalence of 26% [46]. Another systematic review reported prevalence rate of alcohol dependence in prisoners ranging from 18 to 30% in males and 10 to 24% in females [47].

When it comes to low-and middle-income countries, there are very few studies on the prevalence of SUD among prisoners. A recent systematic review by Fazel and colleagues [46], reported that only 2 out of 10 studies included in the review were from low- and middle-income countries. Prevalence of 45 and 30% were reported from those two countries for AUD [46]. When it comes to Africa, very few studies examined SUD among prisoners. One of these is a study from Kenya which reported a prevalence of 65.1% for AUD [11]. In a Nigerian prison study, a prevalence of 13% was reported for past history of alcohol abuse [39].

Substance use before imprisonment is reported to be associated with younger age, male sex, urban residence, higher educational status, physical health problem, type of crime committed (property theft, rape, or fraud), seriousness of crime, repeated offence, and presence of depression [11, 40, 48–51]. Prisoners with history of SUD were found to have twice increased risk of suicide attempt compared to those who did not have SUD [13].

In Ethiopia, a systematic review on the epidemiology of alcohol use reported 24 and 44% pooled prevalence for current and life time alcohol consumption, respectively [52]. None of the included studies in this review had involved prisoners. A study conducted among prisoners in Jimma town who had committed homicide reported a prevalence of 34% for history of alcohol abuse, and 44% for history of khat use [12]. However, AUD was not included in that study. Another study from the same setting also had reported a 40% prevalence for AUD among prisoners [53]. Those studies were conducted in an area which has a different context from Debre Berhan where alcohol production and use is said to be commoner. While, khat (an amphetamine-like psychoactive substance) use is commoner in Jimma.

Huge unmet need, in terms of mental health services for prisoners, was reported in a Nigerian study [54]. Hence, this study can help different stakeholders such as the police, the health sector, and other governmental and non-governmental organizations in designing and implementing strategies to reduce alcohol-related problems including criminality and meet the needs of prisoners through evidence-based interventions. Therefore, this study aimed to examine the prevalence and associated factors of AUD among prisoners 1 year before imprisonment in Debre Berhan Prison (DBP).

## Methods

### Study setting and period

We conducted the study in May 2017, in Debre Berhan, a zonal town of the Amhara Regional State, located 130 km North-East of Addis Ababa, the capital city of Ethiopia [55]. It lies at an altitude of 2830 m above sea level and its annual temperature ranges from 10 C^0^ to 28 C^0^ [56]. In 2015, Debre Berhan town had a population of 102,100 [57].

According to administrative office of the prison, DBP was established in 1941 with around 65 prisoners in an old building. Now the prison has moved to a new building starting 28th September 2015 with 1582 prisoners. In this prison, there were 1933 eligible individuals for our study during the study period. Prisoners get mental health services from Debre Berhan referral hospital, and the prison administration covers the costs for their health services.

### Study design and sampling

We conducted a cross-sectional study among male prisoners in the said prison. Single proportion formula with assumption of 44% prevalence based on the report from Jimma prison study in Ethiopia on SUD [12], 5% point margin of error, 95% confidence interval, and 10% non-response rate was used to determine the sample size for the study. Since the total population of the study was less than 10,000 (*N* = 1933), we applied correction formula (i.e. nf = n/ (1 + n/N)). Using the above formula and assumptions, the final sample size was 347.

We included all male convicted prisoners who were willing to take part in the study and who had stayed in the prison for 5 years or less to minimize recall bias. We excluded female prisoners from this study because there were only 19 of them as it would be difficult to generalize from such small number of respondents.

We used a systematic sampling method to select study participants. Taking the 1933 eligible prisoners as a sampling frame we calculated the “K” value. Our calculated sample size (*n* = 347) gave us a “K” value of six. We obtained prisoners’ list from the attendance sheet and we chose one prisoner from the list (the sampling frame) randomly, using the lottery method of selection. Then, we contacted every 6^th^ prisoner in the list. When we encountered unwillingness to take part in the study, we replaced that participant with the next prisoner from the attendance list. For privacy purpose, we used codes rather than names or other identifiers during data collection. Interviews were conducted in a private room within the prison compound.

### Variables

The outcome variable of this study was AUD, and explanatory variables were socio-demographic factors such as age, occupation, income, religion, and residence; social factors such as physical separation from parents during childhood, experience of history of abuse, satisfaction in life, physical and mental health status, living condition, and family history of substance use. Crime-related information such as year of imprisonment, the reason for imprisonment, previous history of imprisonment, and perception of the relationship between current offence; and substance use history were also collected.

### Measures

#### Assessment of socio-demographic characteristics

Socio-demographic characteristics of the participants were assessed using a structured questionnaire, developed by the researchers, which has 10 items.

#### Assessment of crime-related factors

Criminal background was assessed using a structured questionnaire, developed by the researchers which has six items.

#### Assessment of social factors

Other social factors such as childhood abuse were assessed using a structured questionnaire, developed by the researchers, which has 15 items.

#### Assessment of alcohol use disorder

We assessed AUD using WHO’s Alcohol Use Disorder Identification Test (AUDIT) questionnaire, which has 10 items with four response categories. The total score of AUDIT ranges from 0 to 40. At cut-off point of eight, the tool has shown on average 90% sensitivity and 80% specificity in many countries [58]. AUDIT is a well-evaluated measure where systematic reviews reported remarkable reliability and validity in terms of test-retest reliably, internal consistency, and criterion validity [59, 60]. In this study those who scored eight and above were considered as having AUD. Scores 8 to15 were considered as indicators of harmful drinking, scores 16 to 19 as hazardous drinking, and scores of 20+ as dependency.

To determine a standard ‘drink’, we converted local drinks to grams of pure alcohol, and then we specified the amount of pure alcohol per each local drink and local unit of measure. The level of alcohol content of local drinks was taken from a previous work by Fekadu and colleagues [61]. We considered 10 g of pure alcohol as one standard unit of drink. The details of the calculation can be found from the principal investigator upon request. AUDIT is reported to be suitable for the prison population in non-English speaking communities [62]. The Amharic version of AUDIT was adapted and used in Ethiopia previously to assess AUD in many published works [53, 63–66]. This tool assesses a one-year prevalence of AUD. Since it is not allowed to drink alcohol in DBP, it was not possible to determine current prevalence of AUD. Therefore, we assessed a one-year prevalence before imprisonment.

#### Data collection procedures

We used the adapted Amharic version of AUDIT in this study as in the previous studies. For the exposure variables, we developed and used questionnaires in Amharic, the official language of the country spoken in the study area.

We conducted a pre-test in 17 pre-trial detainees (5% of our main sample) in Debre Berhan police custody, and we made correction on ambiguous items in our tools before the actual data collection. Five trained graduating class BSc nursing students collected data through a face to face interview. A mental health professional supervised the data collection process.

#### Data analysis

We entered the data using EPI-data version 3.1 program and analyzed it using Stata version 13. The data are available and can be supplemented in comma-delimited (*.csv) dataset format, up on request. We checked the data for consistency, outliers, and missing values. We also conducted a reliability analysis of the outcome measure. We used both univariate and multivariate binary logistic regression analyses to identify factors significantly associated with AUD. In the multivariate model, we included variables with *p*-value < 0.05 as independently associated with the outcome variable. We measured the strength of association between the outcome and the exposure variables using odds ratio (OR) with 95% confidence interval (CI).

## Results

### Socio-demographic characteristics of participants

A total of 347 male prisoners took part in the study. The response rate was 100% (*n* = 347). The mean age (in years) of the respondents was 27.76; SD + 11.4 years (Range: 16–82 years). About one-third of the participants (29.1%) were in the age range of 16–20 years. The mean age (in years) of the participants at the time of imprisonment was 26.22; SD + 11.3 years. About half of the participants (48.1%) had primary level education before imprisonment. The median monthly income (in Ethiopian Birr (ETB)) of the participants before imprisonment was 1500 Ethiopian Birr (ETB) (1 USD = 23.09 ETB, during the period of data collection). The vast majority of the participants were from Amhara Ethnic group (93.4%), and followers of Orthodox Christianity (93.1%). About three out of five (59.7%) were single, and a little more than half (51.6%) of the study participants were rural residents (Table 1).

**Table 1:** Socio-demographic characteristics of participants

Variable	Response categories	Frequency (*n* = 347)	Percentage
Age	<= 20	101	29.1
	21–24	76	21.9
	25–29	76	21.9
	≥ 30	94	27.1
Marital status	Unmarried^c^	216	62.2
	Married	131	37.8
Educational status	Unable to read and write	93	26.8
	Primary education	167	48.1
	Secondary education	65	18.7
	Territory education	22	6.3
Occupational status before imprisonment	Government employee	20	5.8
	Personal business	83	23.9
	Daily labourer	27	7.8
	Student	32	9.2
	Farmer	144	41.5
	Other^b^	41	11.8
Ethnicity	Amhara	324	93.4
	Other ^a^	23	6.6
Religion	Orthodox Christian	323	93.1
	Other^d^	24	6.9
Frequency of church/mosque visit before imprisonment	Daily	67	19.3
	2–3 times per week	32	9.2
	Once a week	154	44.4
	Less than weekly	61	17.6
	Never	33	9.5
Residence	Urban	168	48.4
	Rural	179	51.6

^a^Oromo, Tigray, Afar, and Gurage. ^b^Car driver, assistant driver, and a broker. ^c^Single, divorced, separated, and widowed. ^d^ Protestant and Muslim

### Social factors, and the crime history of the participants

As showed in Table 2, a little more than half of the participants (52.7%) stayed in prison for less than 1 year. The reason for imprisonment for almost one-third (29.7%) of the participants was committing homicide. For a vast majority of the respondents (95.4%) this is their first imprisonment. Over a third (34.6%) of the participants reported that they committed the crime under the influence of a substance (Table 2).

**Table 2:** Index crime committed by participants

Variable	Response categories	Frequency (*n* = 347)	%
Index offense	Homicide	103	29.7
	Theft and robbery	67	19.3
	Rape	41	11.8
	Fight	91	26.2
	Others ^a^	45	13.0
Length of stay in prison	≤ 12 months	183	52.7
	> 12 months and < 5 years	164	47.3
Prior history of imprisonment	Yes	16	4.6
	No	331	95.4
Perception that the crime was related to substance use	Yes	120	34.6
	No	227	65.4

^a^ Road traffic accident (RTA), protection of a person who committed crime, smuggling, human trafficking, abduction, corruption, and tax evasion

More than one-fifth of the participants (22.2%) reported that they got separated from their families physically when they were small children; while 11.5% reported experiencing childhood abuse. Only 7.8% of the participants reported that their family had a history of substance use (khat chewing accounting for 63.0% of substances used). One-fifth (19.9%) of the participants used to live alone before the current imprisonment. The majority (83.0%) reported that they had no problem to meet their daily needs or the needs of their families. More than two-thirds of the prisoners rated their overall level of satisfaction in life as good and very good (36.6 and 35.7%, respectively). Most of the study participants reported that they had no major physical or mental illness before imprisonment (90.0, and 95.1%, respectively) (Table 3).

**Table 3:** Social-related characteristics of participants

Variable	Response Categories	Frequency (*n* = 347)	Percentage
Separation from parents during Childhood	Yes	77	22.2
	No	270	77.8
Childhood abuse	Yes	40	11.5
	No	307	88.5
Family history of substance use	Yes	27	7.8
	No	320	92.2
Living arrangement before imprisonment	Alone	69	19.9
	With friend	36	10.4
	With family	242	69.7
Satisfaction with life before imprisonment	Very poor	13	3.8
	Poor	25	7.2
	Neutral	58	16.7
	Good	127	36.6
	Very good	124	35.7
Struggling to meet daily needs before imprisonment	Yes	59	17.0
	No	288	83.0
Major physical illness before imprisonment	Yes	34	10.0
	No	306	90.0
Major mental illness before imprisonment	Yes	17	4.9
	No	329	95.1

### Reliability of the outcome measure

Although the instrument used to assess the outcome variable in this study is a standard measure, we conducted internal consistency reliability analysis and found very good internal consistency of the instrument (α = 0.791). Item-total correlation for items 2 and 3 was near to 0.3; while for the reaming items it was above 0.3. Removing any of the items did not improve the overall internal consistency of the instrument significantly. Therefore, we used all the items of AUDIT in our analyses.

### Prevalence of alcohol use disorder

At a cutoff point of eight, 59.1% (*n* = 205), (95%, CI (53.8–64.2%)) of the respondents had AUD 1 year before their imprisonment. Two-third (66.3%) of those with AUD were harmful drinkers (Table 4).

**Table 4:** One-year prevalence and severity of alcohol use disorder

Variable	Response categories	Frequency (*n* = 347)	%
Alcohol use disorder	Yes	205	59.1
	No	142	40.9
Severity of alcohol use disorder	Harmful drinking (8–15)	136	66.3
	Hazardous drinking (16–19)	27	13.2
	Dependence (20+)	42	20.5

### Factors associated with alcohol use disorder

After controlling for the effects of potential confounding variables, being married (Wald chi-square = 5.86; df = 1; AOR = 0.5, 95% CI = 0.2–0.9; *p* = 0.015), and the index offence of others category (which included road traffic accident (RTA), smuggling goods, participating in human trafficking, etc.) (Wald chi-square = 4.84; df = 1; AOR = 2.6, 95% CI = 1.1–5.9; *p* = 0.028) were significantly associated with AUD. The perception that index offence was alcohol-related (Wald chi-square = 21.72; df = 1; AOR = 4.2, 95% CI = 2.3–7.8; *p* < 0.001), and a family history of substance use (Wald chi-square = 6.71; df = 1; AOR = 8.7, 95% CI = 1.7–44.9; *p* = 0.009) were also significantly associated with AUD. Whereas, increased age was not associated with AUD but the *p*-value was close to level of significance (Wald chi-square = 3.72; df = 1; AOR = 2.3, 95% CI = 1.0–5.3; *p* = 0.054) (Table 5).

**Table 5:** Sociodemographic, personal, and criminal related factors associated with alcohol use disorder

Variable	Response category	AUD caseness		COR (95% CI)	AOR (95% CI)
		No (%)	Yes (%)		
Age	≤ 20	38 (37.6)	63 (62.4)	1.0	1.0
	21–24	36 (47.4)	40 (52.6)	0.7 (0.4, 1.2)	0.6 (0.3, 1.3)
	25–29	34 (44.7)	42 (55.3)	0.7 (0.4, 1.4)	1.1 (0.5, 2.4)
	> 30	34 (36.2%)	60 (63.8%)	1.1 (0.6, 1.9)	2.3 (1.0, 5.3)
Marital status	Unmarried^a^	81 (37.5)	135 (62.5)	1.0	1.0
	Married	61 (46.6)	70 (53.4)	0.7 (0.4, 1.1)	**0.46 (0.2, 0.9)**
Frequency of worship	Daily	30 (44.8)	37 (55.2)	0.8 (0.4, 1.4)	0.6 (0.3, 1.1)
	2–3 times per week	15 (46.9)	17 (53.1)	0.7 (0.3, 1.6)	0.6 (0.2, 1.4)
	Once a week	61 (39.6)	93 (60.4)	1.0	1.0
	Less than weekly	29 (47.5)	32 (52.5)	0.7 (0.4, 1.3)	0.6 (0.3, 1.2)
	Never	7 (21.21%)	26 (78.79%)	2.43 (1.00, 5.96)	1.38 (0.49, 3.88)
Residence	Urban	60 (35.7)	108 (64.3)	1.52 (1.0, 2.3)	1.23 (0.7, 2.2)
	Rural	82 (45.8)	97 (54.2)	1.0	1.0
Index offense	Homicidal	54 (52.4)	49 (47.6)	1.0	1.0
	Stealing & Robbery	20 (29.8)	47 (70.9)	2.59 (1.4, 5.0)	2.3 (1.0, 5.2)
	Rape	15 (36.6)	26 (63.4)	1.9 (0.9, 4.0)	1.1 (0.4, 2.8)
	Conflict	36 (39.6)	55 (60.4)	1.7 (1.0, 3.0)	1.7 (0.9, 3.4)
	Other ^b^	17 (37.8)	28 (62.2)	1.8 (0.9, 3.7)	**2.6 (1.1, 5.9)**
History of imprisonment	Yes	2 (12.5)	14 (87.5)	5.1(1.2, 22.9)	4.5 (0.9, 23.7)
	No	140 (42.3)	191 (57.7)	1.0	1.0
Substance use for crime	Yes	23 (19.2)	97 (80.8)	4.6 (2.8, 7.8)	**4.2 (2.3, 7.8)**
	No	119 (52.4)	108 (47.6)	1.0	1.0
Childhood separation	Yes	18 (23.4)	59 (76.6)	2.8 (1.6, 5.0)	1.8 (0.9, 3.6)
	No	124 (45.9)	146 (54.1)	1.0	1.0
Childhood abuse	Yes	6 (15.0)	34 (85.0)	4.5(1.8, 11.0)	1.9 (0.7, 5.6)
	No	136 (44.3)	171 (55.7)	1.0	1.0
Family hx of substance use	Yes	2 (7.4)	25 (92.6)	9.7(2.3, 41.7)	**8.7 (1.7, 44.9)**
	No	140 (43.8)	180 (56.2)	1.0	1.0
Living status	Alone	25 (36.2)	44 (63.8)	1.4 (0.8, 2.5)	1.0 (0.5, 2.1)
	With friend	9 (25.0)	27 (75.0)	2.4 (1.1, 5.4)	2.3 (0.9, 6.0)
	With family	108 (44.6)	134 (55.4)	1.0	1.0
Satisfaction in life	Very poor	4 (30.8)	9 (69.2)	1.6 (0.5, 5.5)	0.6 (0.1, 3.0)
	Poor	7 (28.0%)	18 (72.0%)	1.8 (0.7, 4.7)	1.2 (0.4, 3.4)
	Neutral	23 (39.7%)	35 (60.3)	1.1 (0.6, 2.0)	0.8 (0.4, 1.6)
	Good	53 (41.7)	74 (58.3)	1.0	1.0
	Very good	55 (44.4)	69 (55.6)	0.9 (0.5, 1.5)	0.6 (0.4, 1.2)
Struggle to meet needs	Yes	17 (28.8)	42 (71.2)	1.9 (1.0, 3.5)	1.4 (0.6, 2.9)
	No	125 (43.4)	163 (56.6)	1.0	1.0

*Abbreviations: AOR* Adjusted odds ratio, *AUD* Alcohol use disorder, *CI* Confidence interval, *COR* Crude odds ratio, *hx* History

Bold is for variables with *p*-value of < 0.05

^a^Single, divorced, separated, and widowed. ^b^road traffic accident, protection of a person who committed a crime, smuggling, illegal person transfer, abduction, corruption, and cheating

The odds of those who were married to be cases of AUD were 50% less compared to unmarried participants (Wald chi-square = 5.86; df = 1; AOR = 0.5, 95% CI = 0.2–0.9; *p* = 0.015). The odds of those who were imprisoned because of RTA, contraband, corruption, etc. in having AUD were nearly 3 times higher than those imprisoned because of homicide (Wald chi-square = 4.84; df = 1; AOR = 2.6, 95% CI = 1.1–5.9; *p* = 0.028). Furthermore, the odds of prisoners who perceive that their current offence was related to substance use in having AUD were about 4 times higher than those who did not think that substance use were not related to their current offence (Wald chi-square = 21.72; df = 1; AOR = 4.2, 95% CI = 2.3–7.8; *p* < 0.001). The odds of those who had family history of substance use in having AUD were 9 times higher compared to those who did not report family history of substance use (Wald chi-square = 6.71; df = 1; AOR = 8.7, 95% CI = 1.7–44.9; *p* = 0.009) (Table 5).

## Discussion

This study found that around six out of ten prisoners (59.1%) had AUD 1 year before imprisonment. This finding is consistent with a study conducted among prisoners in England, where 63.0% of male prisoners were found to have AUD [35]. On the other hand, our study showed a much higher prevalence compared to many of the previous reports from different parts of the world which reported prevalence rates ranging from 13.7 to 51.0% [16, 20, 31–33, 38, 40, 41, 43, 46, 47]. Our finding is also higher compared to a previous prison based studies in Ethiopia which reported a 34.0% [12], and 40.1% [53] prevalence for alcohol use and AUD, respectively. Our finding is also much higher compared to the results of a meta-analysis on the epidemiology of alcohol use in Ethiopia which reported a 24% pooled prevalence of current use [52]. We had expected that the prevalence of AUD in our study would be lower than those previous findings of use and abuse which one would think are commoner and less problematic compared to AUD. The possible explanation for this finding may be that production and usage of both local and industrially manufactured alcoholic drinks is higher in Debre Berhan compared to those places where other studies were conducted. Debre Berhan is much colder in its weather than those other places and it is also culturally acceptable to drink alcohol to warm up and overcome the effects of the freezing weather. Furthermore, majority of the participants in this study were Orthodox-Christian followers in which drinking alcohol is not prohibited by the religion. On the other hand, the prevalence of AUD in our study is lower than some of the previous studies which reported prevalence ranging from 69.3 to 73.0% [11, 34, 42]. This variation may be due to several reasons, including differences in socio-cultural, economic, and living conditions. The difference in methodology and measures used might also have contributed to the difference in the findings.

In this study the odds of those who were unmarried were two times higher to be cases of AUD compared to married prisoners. Previous population-based studies have found that AUD is associated with being unmarried [67–70]. The possible explanation for this finding may be that married life is said to bring a more stable lifestyle and responsibility for the family which may not allow people to spend their income on drinking alcohol. Once they are married, they may not have the financial ability to meet the needs of their family as well as for the drink.

The increased association between AUD and the type of crime committed in this study is also similar to previous studies which found that alcohol use is associated with type of crime committed such as property crime [27, 40] and rape [51]. The higher association between AUD and participants perception that their current offense was related to substance use may have similarity with a findings that reported association between substance and crime; and intoxication and crime [9, 45, 50].

Increased association between AUD and family history of substance use seen in this study is consistent with other reports from general population studies [70–72]. This can possibly be explained by genetic and environmental predispositions of the participants which has been well described in the literature. Substance use is a behaviour that one can learn from his/her exposure to family or community environment that commonly use or have appreciation for substance use.

To our knowledge, this study is one of the very few studies in the Ethiopian prisoners to investigate the prevalence and associated factors of alcohol related problems using a standardized instrument. Nevertheless, readers need to consider the following limitations while interpreting the findings. First, we conducted the study only among male prisoners because there were only 19 female prisoners during the study period. Second, there might be recall bias, because participants were asked about their experience with alcohol drinking 1 year before they were imprisoned. The other limitation of the study is social desirability bias. Respondents might have undermined or increased the amount of alcohol consumption they have reported as data collection was done using interview method. Finally, it is likely that Type I error may have been introduced while performing bivariate analyses due to multiple testing.

This study has implication to reduce AUD in the community and prevent its adverse effects such as committing crime and for planning appropriate intervention for people with this problem in the prisons.

## Conclusions

In this study we found that prevalence of AUD 1 year before imprisonment to be very high. More than one-third (34.6%) of the prisoners reported that they committed the current crime under the influence of a substance, and this is significantly associated with AUD. This suggests that substance use may increase the risk of criminality. However, this may not be necessarily true and one cannot establish such conclusion from a cross-sectional study. It would be more appropriate to conduct a follow-up study which allows us to establish the temporal relationship between substance use and criminality.

Being not married before imprisonment; type of index crime such as RTA, contraband, abduction, and others; perceiving that substance use is related to the current offence; and having a family history of substance use were significantly associated with AUD. These findings call for appropriate actions such as creating awareness about consequences of AUD, employing AUD prevention strategies, providing early detection and intervention services, and making AUD treatment available and accessible both in the community and in the prison.

Therefore, a collaborative action needs to be taken by different stakeholders (the police, the health sector, and other government and non-governmental organizations), on the prevention and treatment of AUD in the community and in the prison population. Since there is lack of research on AUD and crime in Ethiopia, we recommend future studies on the subject matter.

## Data Availability

The data used for this study can be made available with a reasonable request from the corresponding author.

## Abbreviations

AOR: Adjusted odds ratio
AUD: Alcohol use disorder
AUDIT: Alcohol use disorder identification test
CI: Confidence interval
DALYs: Disability-adjusted life years
DBP: Debre Berhan Prison
OR: Odds ratio
RTA: Road traffic accident
SUD: Substance use disorder
WHO: World Health Organization
YLDs: Year’s lost due to disability
